# Physical Activity and Sedentary Behavior among Young Adolescents in 68 LMICs, and Their Relationships with National Economic Development

**DOI:** 10.3390/ijerph17217752

**Published:** 2020-10-23

**Authors:** Chuanwei Ma, Yuanyuan Zhang, Min Zhao, Pascal Bovet, Bo Xi

**Affiliations:** 1 Department of Epidemiology, School of Public Health, Cheeloo College of Medicine, Shandong University, Jinan 250012, China; chuanwei_ma@126.com; 2 Women’s Hospital, Zhejiang University School of Medicine, Hangzhou 310006, China; yuanyuanzhang2010@126.com; 3 Department of Nutrition and Food Hygiene, School of Public Health, Cheeloo College of Medicine, Shandong University, Jinan 250012, China; zhaomin1986zm@126.com; 4 Center for Primary Care and Public Health (Unisanté), University of Lausanne, 1010 Lausanne, Switzerland; pascal.bovet@unisante.ch

**Keywords:** physical activity, sedentary behaviors, adolescents, low- and middle-income countries, purchasing power parity

## Abstract

It is unclear whether physical activity and sedentary behavior are associated with economic development in low- and middle-income countries (LMICs). We aimed to assess the association between these two behaviors and country economic development among young adolescents in LMICs. Data came from the Global School-based Student Health Survey (GSHS) conducted between 2009 and 2016 in 68 LMICs. A total of 180,298 adolescents aged 12–15 years were included; 15.3% of young adolescents achieved the recommended level for sufficient physical activity (≥60 min/day of physical activity of any kind per week according to WHO) and 64.6% achieved a low sedentary behavior (≤2 h of sitting activities/day according to some guidelines, not accounting for sitting time at school or for doing homework). However, only 9.1% of young adolescents met the recommended levels of both behaviors. Comparing the lowest to the highest quintiles of a country’s purchasing power parity per capita (PPP), mean values of both physical activity (boys: 2.55 to 2.96 days/week; girls: 2.10 to 2.31 days/week) and sedentary behavior(boys: 1.86 to 3.13 h/day; girls: 1.83 to 3.53 h/day) increased. The prevalence of having both recommended behaviors decreased among boys (12.0% to 10.0%) and girls (9.6% to 4.9%) (*p* < 0.001). Although there might be an ecological fallacy, the findings emphasize the need for interventions to increase physical activity and reduce sedentary behavior among children and young adolescents.

## 1. Introduction

Non-communicable diseases (NCDs) are the leading cause of mortality worldwide, including in low- and middle-income countries (LMICs) [1,2]. Physical inactivity and sedentary behavior are well-known risk factors for NCDs. It was estimated that lack of physical activity could contribute up to 6–10% of the NCD morbidity (e.g., coronary heart disease, type 2 diabetes, and breast and colon cancers), and 9% of NCD premature mortality [3]. It has been estimated that physical inactivity in LMICs is responsible for 75.0% of the 13.4 million disability-adjusted life years globally [4], and life expectancy could increase by 0.68 years if physical inactivity was eliminated [3].

In youths, physical activity is beneficial to metabolic health in many ways, including the reduction of abdominal fat, blood pressure, blood glucose, and arterial stiffness, and increase in blood HDL cholesterol [5]. In addition, regular physical activity during childhood has a favorable effect on several outcomes in adulthood, including reduction of the risk of cardiovascular disease [6,7]. One previous review of the Global School-based Health Survey (GSHS) conducted between 2003 and 2007 in 34 countries showed that only a minority of adolescents (23.8% of boys and 15.4% of girls) met the World Health Organization (WHO) physical activity target (≥60 min/day), and more than one third of children engaged in sedentary behavior for ≥2 h per day (e.g., watching television or playing computer games) [8]. Based on the GSHS in 2001 to 2016, another study reported that ≥80% of students aged 11–17 years were physically inactive [9]. However, physical inactivity and sedentary behavior can have detrimental effects on health independently of each other [10], and information on sedentary time was not included in this study. A review of 130 surveillance studies showed ≥50% of children and adolescents engaged in sedentary behavior for ≥2 h/day in recent years [11]. Based on the GSHS in 2006–2016, it was estimated that 26.4% of adolescents had sedentary behavior for ≥3 h/day during their leisure time [12].

Member states of WHO agreed on a global target of a 10% relative reduction in physical inactivity between 2010 and 2025 for the prevention and control of NCDs [13]. In addition, WHO has developed a new global plan for physical activity in youth, which includes enhancing physical education and school-based programs, walking and cycling to school programs, and improving access to public open spaces, among several other measures [14]. Therefore, it is important to regularly update estimates on the prevalence of physical activity and sedentary behavior in children and adolescents in all countries in order to monitor and guide effective interventions to increase physical activity and decrease sedentary behavior.

There is a relationship between the socioeconomic status of a country and levels of physical activity [15] and sedentary behavior [16]. For example, Wang et al. found that people tended to engage in more physical activity in economically advanced regions in China, and the correlation coefficient between physical activity and gross domestic product (GDP) per capita was 0.23 for men and 0.15 for women [15].A systematic review and meta-analysis including 39 countries found that the prevalence of physical inactivity differed according to country GDP per capita. For example, the prevalence increased with GDP per capita in LMICs, but decreased in high-income countries [16]. However, Guthold et al. did not find a consistent pattern of physical inactivity according to country income (low, low-middle, upper-middle, and high level) [9]. Furthermore, the socioeconomic status of a country was assessed based on the GDP per capita in most studies. However, the purchasing power parity (PPP) per capita may be a better indicator of a country’s economic development than GDP per capita, because PPP is adjusted for the living costs and inflation of a country [17].

Therefore, using the most recent GSHS data, we assessed the prevalence of physical activity and sedentary behavior, and their associations with a country’s economic development (measured with PPP/capita) in young adolescents aged 12–15 years in 68 LMICs.

## 2. Methods

### 2.1. Study Design and Participants

The GSHS was developed by the WHO and the U.S. Centers for Disease Control and Prevention (CDC) [18,19]. The GSHS aims to assess the health behavior of young adolescents aged 12–15 years in LMICs to help countries develop health programs and policies. The survey uses a self-administered standard questionnaire to assess health behavior in random samples of school-going young adolescents during regular school hours. The questionnaire was jointly created by the WHO and U.S. CDC and translated into the local languages for each country.

We obtained the most recent GSHS datasets between 2009 and 2016 from the WHO’s website [18]. We categorized the location of each country according to the WHO region. Briefly, in each country, a standardized two-stage cluster sampling design was applied to obtain a nationally representative sample of young adolescents. In the first stage, schools were randomly selected by the probability proportional to size sampling. In the second stage, classes were randomly selected in the selected schools. All students in the selected classes were eligible to participate in the survey, and the participation was anonymous and voluntary. To directly compare estimates between countries, the wording of the core questions could not be altered in GSHS. All GSHS surveys in each country were approved by the Ministry of Education and an institutional review board or ethics committee. Verbal or written consent was obtained from all students and their parents/guardians.

### 2.2. Definitions of Physical Activity and Sedentary Behavior

The definitions of physical activity and sedentary behavior were based on the corresponding questions in the questionnaire (https://www.who.int/ncds/surveillance/gshs/GSHS_Core_Modules_2013_English.pdf).

Physical activity was assessed using the question: “During the past seven days, on how many days were you physically active for a total of at least 60 min per day?” (Add up all the time you spent in any kind of physical activity each day). The corresponding response options included “0 days”, “1 day”, “2 days”, “3 days”, “4 days”, “5 days”, “6 days”, and “7 days”. Physical activity was defined as any activity that increases a student’s heart rate and makes the student out of breath some of the time. Physical activity includes leisure time sports, exercise played with friends, or walking to school. Some examples of physical activity include running, fast walking, biking, dancing, and playing football, etc. In our analysis, sufficient physical activity was defined as meeting the WHO recommendation of ≥60 min/day of moderate to vigorous intensity physical activity of any kind during the past seven days [20]. The assessment of physical activity levels was tested to have good reliability and validity [21].

Sedentary behavior was assessed using the question: “How much time do you spend during a typical or usual day sitting and watching television, playing computer games, talking with friends, or doing other sitting activities (after excluding sitting time at school and at home for homework)?” The corresponding response options included “less than 1 h per day”, “1 to 2 h per day”, “3 to 4 h per day”, “5 to 6 h per day”, “7 to 8 h per day”, and “more than 8 h per day”. We calculated the mean values of sedentary behavior (hours/day)after transforming the responses as follows: “less than 1 h per day” was coded as “0.5 h”, “1 to 2 h per day” as “1.5 h”, “3 to 4 h per day” as “3.5 h”, “5 to 6 h per day” as “5.5 h”, “7 to 8 h per day” as “7.5 h”, and “more than 8 h per day” as “8.5 h”. Because the Canadian 24-h Movement Guidelines recommend no more than 2 h/day of screen or sitting time for children aged 5–17 years [22], we considered high sedentary behavior in our study as ≥2 h per day spent on sitting activities (beyond sitting time at school or for doing homework).

We used PPP/capita as the socioeconomic indicator of a country’s economic growth [23]. We used PPP per capita data from the World Bank (60 countries) and the Index Mundi (eight countries) that corresponded to the survey year of the GSHS in our study [24]. PPP/capita is adjusted for inflation and living costs of a particular country, which may therefore enable better comparison of results between countries as compared to GDP/capita. To examine the effect of economic development on physical activity and sedentary behavior, a country’s PPP/capita was classified into five quintiles in our study (Q1: $100–$4299; Q2: $4300–$7799; Q3: $7800–$15,999; Q4: $16,000–$22,999; Q5: >$23,000).

### 2.3. Statistical Analysis

We calculated prevalence and mean estimates using strata, primary sampling units, and sampling weights at the country level in consideration of the complex sampling design of the GSHS. We considered a difference according to sex or region to be statistically significant if the 95% confidence intervals (95% CIs) did not overlap, except for results from the logistic regression analysis. Estimates of prevalence and mean values of physical activity and sedentary behavior according to region were calculated using meta-analysis with a random effects model (using STATA version 11.0) because of significant between-study heterogeneity. Linear regression was used to assess the associations of a country’s PPP/capita with physical activity or sedentary behavior after adjustment for sex, age, and survey year, using the complex samples module in SPSS version 18.0. Logistic regression was performed to assess the associations of a country’s PPP/capita with sufficient physical activity and low sedentary time after adjustment for the considered potential covariates.

## 3. Results

Table S1 shows the characteristics of the surveys and participants. Data came from 68 countries in 5 WHO regions (Africa 9; America 19; Eastern Mediterranean 15; Southeast Asia 5; Western Pacific 20) and were collected between 2009 and 2016. In total, 180,298 young adolescents aged 12–15 years were included in our study. The overall response rate was 96.5%, ranging from 85.4% in Samoa to 99.5% in Laos. The sample sizes ranged from 78 in Niue to 20,416 in Argentina, with a median sample size of 1752. Adolescents were active for ≥1 h/day on 2.48 days/week (95% CI 2.36–2.61), and the estimates were lowest in Cambodia (1.42, 1.32–1.53) and highest in Bangladesh (4.05, 3.75–4.35). Adolescents engaged in sedentary behavior on 2.48 h/day (95% CI 2.30–2.67), and estimates were lowest in Pakistan (1.11, 1.04–1.18) and highest in Kuwait (3.97, 3.65–4.30). The overall prevalence of adolescents having sufficient physical activity was 15.3%, and estimates were lowest in Cambodia (6.5%) and highest in Bangladesh (41.4%). The overall proportion of adolescents having low sedentary time was 64.6%, and estimates were lowest in Barbados (34.9%) and highest in Pakistan (91.8%).

### 3.1. Physical Activity and Sedentary Behavior by Sex, WHO Region, and PPP Category

In the America and East Mediterranean regions and in three of the five PPP/capita categories (except for Q1 and Q3), boys spent more time than girls in physical activity. However, the mean time of sedentary behavior did not differ significantly according to sex, WHO region, and PPP categories (Table 1). Duration of physical activity did not differ significantly according to WHO regions and sex. Mean duration (hours/day) of sedentary behavior among both sexes was lowest in Southeast Asia (boys: 1.89, 1.38–2.40; girls: 1.82, 1.26–2.37) and highest in America (boys: 2.80, 2.53–3.07; girls: 3.07, 2.75–3.39). With increasing PPP/capita, mean duration of physical activity decreased in the lower PPP quintiles and increased in the upper PPP quintiles, while the mean duration of sedentary behavior increased gradually according to PPP/capita in boys and girls (*p* for trends < 0.05) (Table 1).

### 3.2. Country PPP and Association with Physical Activity and Sedentary Behaviors

**Figure 1:**
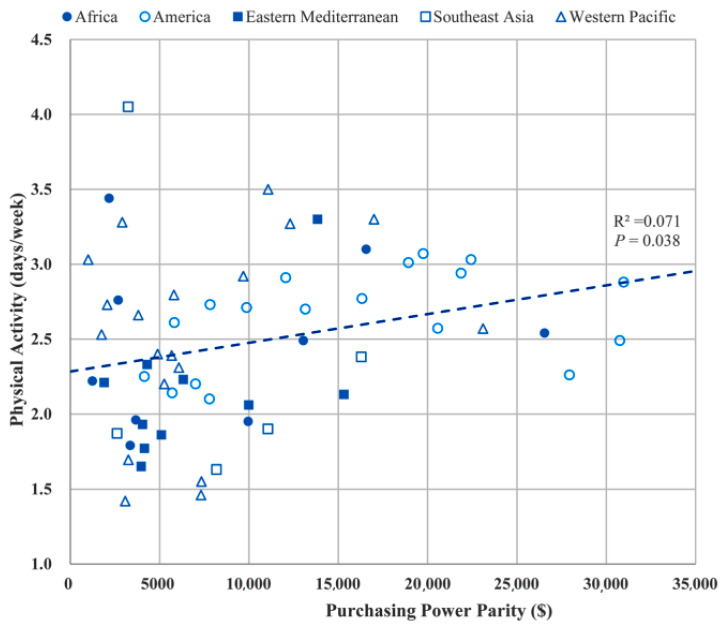
Mean daily duration of physical activity of any kind by purchasing power parity per capita in 68 LMICs and 5 WHO regions.

**Figure 2:**
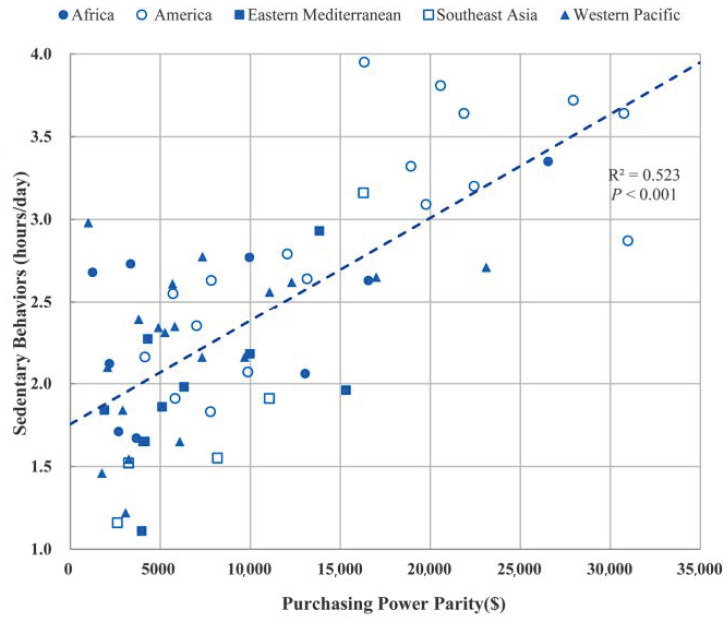
Mean daily duration of sedentary behavior (except for sitting time at school or for homework) by purchasing power parity per capita in 68 LMICs and 5 WHO regions.

Figure 1 and Figure 2 show the distribution of mean duration of physical activity (days with ≥1 h/day) in the past week and the mean duration (hours/day) of sedentary behavior, respectively, by a country’s PPP/capita in the 68 LMICs and in the 5 WHO regions. Linear regression showed that PPP/capita was positively associated with the number of days with sufficient physical activity and duration of sedentary behavior per day, respectively, after adjustment for sex, age, and survey year (Table 2). After adjustment for physical activity to sedentary behavior, and vice versa, the association of PPP/capita with the number of days with sufficient physical activity or sedentary behavior remained (data not shown).

The prevalence of sufficient physical activity was higher in boys than in girls in the America (21.0% vs. 12.4%), East Mediterranean (17.8% vs. 11.0%), and Western Pacific (17.1% vs. 11.7%) regions, and the prevalence of sufficient physical activity was higher in boys than girls in the four upper PPP quintiles than in the first one ($100–$4799). The prevalence of sufficient physical activity did not differ significantly according to WHO regions in both sexes (Table 3). There were not significant differences in the prevalence of low sedentary time according to sex across all WHO regions and PPP/capita categories. The prevalence of low sedentary time was lowest in America (boys: 57.4%, 95% CI 52.0–62.9; girls: 52.5%, 46.2–58.8) and highest in Southeast Asia (boys: 76.4%, 66.1–86.7; girls: 78.0%, 65.8–90.2). The prevalence of sufficient physical activity decreased largely (24.7% to 12.7%) along the first two quintiles of PPP/capita and increased across the upper quintiles gradually (12.7% to 19.9%) among boys, while there was a downward trend across the five PPP/capita quintiles among girls (19.6% to 9.5%). The prevalence of low sedentary time decreased gradually according to the five PPP quintiles among both sexes (boys: 83.7% to 56.6%; girls: 84.3% to 53.4%) (Table 3).

Compared to the second quintile of PPP, the odds of having sufficient physical activity were larger in the upper PPP/capita categories (Q3: OR= 1.31, 95% CI = 1.15–1.50; Q4: OR = 1.39, 95% CI = 1.20–1.61; Q5: OR = 1.52, 95% CI = 1.34–1.73) after adjustment for sex, age, and survey year (Table 4). In addition, compared to the first PPP/capita quintile, the odds of having low sedentary time were also higher in the upper PPP/capita categories (Q2: OR = 0.44, 95% CI = 0.38–0.51; Q3: OR = 0.51, 95% CI = 0.44–0.60; Q4: OR = 0.18, 95% CI = 0.16–0.21; Q5: OR = 0.23, 95% CI = 0.20–0.26) (Table 4).

### 3.3. Country PPP per Capita and Its Association with Combined Insufficient Physical Activity and/or High Sedentary Behavior

The overall prevalence of having both sufficient physical activity (≥1 h per day) and low sedentary time (≤2 h per day) was only 9.1%, and was lowest in the Philippines (3.4%) and highest in Bangladesh (35.0%) (Table S2).The prevalence of having sufficient physical activity and low sedentary behavior was higher in boys than in girls in Africa (12.1% vs. 7.3%), America (11.2% vs. 6.1%), and East Mediterranean(10.5% vs. 6.6%) regions, as well as in the last three upper PPP/capita quintiles (Q3, 12.6% vs. 8.1%; Q4, 11.5% vs. 5.3%; Q5, 10.0% vs. 4.9%), but did not differ significantly across WHO regions(Table 5). The prevalence of having insufficient physical activity and high sedentary behavior (>2 h per day) did not differ according to sex, WHO region, or PPP/capita categories (except for Q4, boys vs. girls: 37.7% vs. 48.0%), while it was the highest in America (boys: 32.7%, girls: 41.1%) and the lowest in Southeast Asia (boys:18.7%, girls:19.3%) (Table 5). The prevalence of having sufficient physical activity and low sedentary behavior and the prevalence of having insufficient physical activity (<1 h/day) and low sedentary behavior (≤2 h/day) decreased along increasing PPP/capita quintiles among both sexes, while there were upward trends with increasing PPP/capita quintiles among both sexes in the other two groups (Table 5).

Compared to countries with the lowest quintile of PPP, young adolescents in countries with higher quintiles of PPP were less likely to have both sufficient physical activity and low sedentary time (Q2: OR = 0.30, 95% CI = 0.28–0.36; Q3: 0.42, 0.36–0.50; Q4: 0.34, 0.29–0.41; Q5: 0.40, 0.34–0.47) after adjustment for sex, age, and survey year, whereas odds ratios for not having both sufficient physical activity and low sedentary time increased along PPP/per capita quintiles (Q2: 2.53, 2.22–2.89; Q3: 2.08, 1.82–2.39; Q4: 5.74, 4.93–6.67; Q5: 4.58, 4.08–5.14) (Table 6).

## 4. Discussion

Overall, the prevalence of both sufficient physical activity and low sedentary behavior was relatively low among young adolescents aged 12–15 years in LMICs. There was an upward trend for both physical activity and sedentary behavior along increasing country PPP/capita quintiles. In addition, the relation to country PPP/capita quintiles(i.e., analysis done with countries as unit) showed that the prevalence of sufficient physical activity (≥1 h per day) decreased between the first and second PPP/capita quintiles and then increased along increasing PPP/capita quintiles (i.e., a J-curve shape). The prevalence of low sedentary time decreased with the increase of PPP quintiles among both sexes.

Previous studies have reported a low prevalence of sufficient physical activity in young adolescents worldwide [9]. In 2011, a study using data from 105 countries showed that only 19.7% adolescents met the recommended level of ≥60 min/day of physical activity [25], which is similar to our results. Steene-Johannessen et.al. found that only 29% of children and adolescents aged 2–18 years were sufficiently physically active (using the same criterion of ≥60 min/day) among European countries [26]. Another previous study using the GSHS data from 34 countries between 2003 and 2007 showed that, among adolescents aged 13–15 years, 23.8% of boys and 15.4% of girls met the recommendation of sufficient physical activity [8], which is consistent with our findings that boys are more active than girls. The sex difference might be due to environmental factors and gender norms. For example, unlike boys, girls’ outdoor physical activity can be associated with street violence [27], and passive road safety was significantly associated with the increase of physical activity among girls [28]. It seemed that girls may receive more parental restrictions for engaging in exercise, and exercise by girls may be more easily affected by environmental factors [29]. In addition, the road environment (e.g., traffic/pedestrian lights, residing on a cul-de-sac) could influence adolescents’ physical activity [30]. These findings suggest that global gender specific strategies on environmental barriers could improve physical activity among adolescents.

Few studies have assessed the prevalence of sedentary behavior in young adolescents using global data, and little is known about this question in LMICs. Our findings suggest a high prevalence (35.4%) of sedentary behavior (>2 h/day) among young adolescents aged 12–15 years in LMICs, which is consistent with a review based on 130 surveillance studies [11]. A recent meta-analysis showed that 3–4 h/day of TV reviewing was associated with a greater risk of all-cause, cardiovascular, and cancer mortality, as well as incident type 2 diabetes [31]. Rees-Punia et al. reported that the risk of premature death could be reduced if the amount of sitting time was replaced by physical activity, even moderate, and the benefits would be larger if sedentary behavior was replaced with moderate to vigorous physical activity [32].

We also found that less than 10% of adolescents overall met both recommendations on sufficient physical activity and low sedentary behavior. Because low physical activity and high sedentary behavior have independent adverse effects on health [33,34], adolescents with both insufficient physical activity and sedentary behavior may suffer from amplified adverse health outcomes. In addition, we found that over 50% of adolescents who had low sedentary behavior had insufficient physical activity, suggesting that adolescents who have low levels of sedentary behavior can have insufficient physical activity. One previous meta-analysis showed that there was only a small inverse association between physical activity and sedentary behavior among children and adolescents, emphasizing that the two behaviors do not directly necessarily correlate well [35]. Our findings suggest the need to promote sufficient physical activity among adolescents, including for those who do not have sedentary behavior, and vice versa.

Several previous studies have examined the association between a country’s economic status and physical activity using a national GDP per capita with inconsistent findings [36,37,38]. Data from European countries have demonstrated that country GDP/capita was positively associated with levels of physical activity [36], although other studies showed that high-income countries have a large prevalence of adolescents with insufficient physical activity [37]. Consistent with this latter study, we found that country economic level, assessed by the country’s PPP/capita, was positively associated with the number of days with sufficient physical activity in young adolescents in LMICs [35].

When the distribution of physical activity is analyzed within a particular country (i.e., not between countries), based on the GSHS conducted from 2009 to 2016, Vancampfort et al. found that adolescents from poor families were less likely to meet the recommended sufficient physical activity [39].This question was not assessed in the present study, as we did not have socio-economic indicators at the individual level for all students included in this study. However, it is known, for example, that students from families with high socio-economic status are more likely to attend gym memberships and to live in a favorable environment, like an area with green areas, sports facilities, bike trails, and adequately connected streets [12].

We also observed in our study among LMICs that young adolescents were more likely to have high sedentary behavior in countries with high than low PPP/capita. Adolescents from the more developed countries (i.e., those with the highest PPP/capita) are likely to have easier access to the internet, computer games, and TV than those from less developed countries (lower PPP/capita). A recent systematic meta-analysis showed an inverse association between socioeconomic status and sedentary behavior in high-income countries but a direct association in LMICs [16]. Moreover, a Brazilian study showed that about 57.3% of adolescents had high screen time, and screen time was positively associated with the socioeconomic status of the adolescents [40]. Altogether, these observations in the literature and our findings suggest that, although the economic development of a country (among LMICs) maybe associated with a greater level of physical activity among adolescents, increased country development may also be associated with adverse health effects due to increased sedentary behavior according to an individual’s socioeconomic status. We also found that, although physical activity increased along the upper PPP/capita quintiles, the prevalence of adolescents with sufficient physical activity tended to decrease within the first and second PPP/capita quintiles, suggesting a J-shape relation.

Economic development at the country level may also alter people’s lifestyles. The weakly positive association of a country’s PPP/capita with the level of physical activity among adolescents and the inverse association between a country’s PPP and sedentary behavior suggest that LMICs with higher PPP/capita should specifically address interventions to reduce sedentary behavior, even though adolescents in these countries may have more physical activity. In addition, LMICs with low PPP/capita should develop interventions to promote physical activity despite adolescents in these countries tending to have less sedentary behavior. Over all, it is important to develop specific strategies to promote physical activity and reduce sedentary behavior among adolescents in LMICs.

Strategies to promote physical activity and reduce sedentary behavior should target people of all ages and genders, in different settings, and be performed through interventions involving multiple sectors [41]. The global action plan on physical activity 2018–2030 set four strategic objectives to increase physical activity and reduce sedentary behavior, including creating active societies, creating active environments, creating active systems, and encouraging people to be active [14]. This can include measures that promote active commuting to school (e.g., walking or cycling), setting a minimum number of hours of physical activity in school curricula, promoting sports for all at leisure time, programs of physical activity in communities and in other settings, and developing green areas and sport premises. Of note, many interventions promoting physical activity can have benefits that extend beyond health, such as improving social cohesion or benefiting the local economic actors, which may further accelerate their implementation. It is also important to regularly evaluate the impact of physical activity interventions and policy. A recent systematic review reported that only 69 of 292 intervention studies mentioned a proper evaluation framework, limiting their potential to be further sustained or implemented elsewhere [42].

### Study Strengths and Limitations

This study had two main strengths. First, it included a large sample of participants from many LMICs in several regions, which strengthens the generalizability of the findings to young adolescents. Second, the same questionnaire was used in all countries, making the results directly comparable across all countries. However, several limitations should also be noted. First, physical activity and sedentary behavior were self-reported, which is far less accurate than objective measurements, such as accelerometers or energy expenditure assessed with doubly labeled water. This may lead to biases toward both over- or underestimation. However, objective measurements of physical activity and sedentary behavior (e.g., accelerometers) are quite resource intensive, making such measurements difficult to use in large epidemiological surveys. Second, sedentary behavior (e.g., sitting time, standing time, and lying time) is difficult to assess precisely by a questionnaire, and could provide more accurate data and would be measured more accurately with objects such as accelerometers, including in smartphones. Cut-offs for sedentary time associated with detrimental health outcomes (e.g., ≥2 h in our study, excluding sitting time at school or for homework) are still quite arbitrary and less validated than physical activity cut-offs associated with detrimental health outcomes. It is also not fully clear to what extent sedentary behavior and insufficient physical activity are independent causes of health outcomes, and whether a person can compensate sedentary time by having more physical activity and vice versa. Third, the comparison of estimates between WHO regions and countries should be interpreted with caution, because surveys were not done at the same time (2009 to 2016). However, the majority of surveys (54 of 68 countries) were performed between 2011 and 2016. Fourth, although some potentially confounding factors were adjusted in a multivariate analysis, residual confounding or unmeasured factors might have influenced our findings. In addition, despite the same wording, questions on physical activity and sedentary behavior (both cutting across many social and behavioral dimensions) may be understood or interpreted differently according to a person’s country, culture, sex, or social background. Fifth, the ecological design of our study (the correlation between PPP/capita at the country level and physical activity and sedentary behavior levels at the individual level) might lead to ecological fallacy.

## 5. Conclusions

Our study shows a relatively low prevalence of sufficient physical activity and relatively high prevalence of sedentary behavior among young adolescents aged 12–15 years in LMICs. Physical activity and sedentary behavior differed according to sex, age, country, and PPP/capita. Further studies should account for a student’s socio-economic status, in addition to adjustment for the country’s economic level, to better guide policy on physical activity and sedentary behavior among adolescents. Notwithstanding methodological limitations of our study, particularly the potential ecological fallacy and the use of a self-administered questionnaire vs. objective measurements of physical activity, our findings emphasize the need to strengthen multi-sectoral interventions and programs to promote regular physical activity on all days of the week and reduce sedentary behavior in young adolescents in LMICs.

## Figures and Tables

**Table 1:** Distribution of number of days/week with sufficient physical activity and number of hours/day with sedentary behavior in adolescents aged 12–15 years by sex, WHO region, and PPP category.

	Days/Week with Sufficient Physical Activity				Hours/Day with Sedentary Behavior			
	Boys		Girls		Boys		Girls	
	N	Mean (95% CI)	N	Mean (95% CI)	N	Mean (95% CI)	N	Mean (95% CI)
WHO region								
Africa	7501	2.78 (2.33–3.23)	9053	2.12 (1.83–2.42)	7501	2.33 (1.98–2.68)	9053	2.46 (2.02–2.90)
America	24,700	2.99 (2.79–3.19)	27,648	2.30 (2.17–2.44)	24,700	2.80 (2.53–3.07)	27,648	3.07 (2.75–3.39)
East Mediterranean	22,175	2.65 (2.40–2.90)	23,334	2.01 (1.76–2.25)	22,175	2.35 (1.91–2.79)	23,334	2.45 (2.01–2.89)
Southeast Asia	9490	2.53 (1.92–3.14)	11,620	2.16 (1.64–2.68)	9490	1.89 (1.38–2.40)	11,620	1.82 (1.26–2.37)
Western Pacific	21,156	2.71 (2.39–3.03)	23,621	2.31 (2.03–2.59)	21,156	2.26 (2.00–2.52)	23,621	2.28 (1.99–2.58)
PPP/capita, $								
Q1 100–4299	14,834	2.55 (2.24–2.87)	15,236	2.10 (1.82–2.38)	14,834	1.86 (1.63–2.09)	15,236	1.83 (1.61–2.04)
Q2 4300–7799	18,684	2.40 (2.23–2.57)	22,647	1.93 (1.77–2.10)	18,684	2.16 (1.94–2.37)	22,647	2.19 (2.05–2.33)
Q3 7800–15,999	15,584	2.87 (2.50–3.24)	17,837	2.30 (1.97–2.64)	15,584	2.33 (2.13–2.52)	17,837	2.33 (2.06–2.60)
Q4 16,000–22,999	17,321	3.34 (3.08–3.59)	19,968	2.49 (2.31–2.68)	17,321	3.10 (2.89–3.31)	19,968	3.40 (3.08–3.72)
Q5 > 23,000	18,599	2.96 (2.79–3.14)	19,588	2.31 (2.16–2.46)	18,599	3.13 (2.92–3.34)	19,588	3.53 (3.19–3.86)
Linear trend across quintiles		<0.001		<0.001		<0.001		<0.001

Adjusted for sex, age, survey year, and country. WHO, World Health Organization; PPP/capita, purchasing power parity/capita. Sufficient physical activity: any physical activity ≥60 min/day; sedentary behavior: sitting time/day except at school or for homework.

**Table 2:** Predictors of number of days/week with sufficient physical activity and of number of hours/day with sedentary behavior in adolescents aged 12–15 years.

	Days/Week with Sufficient Physical Activity			Hours/Day with Sedentary Behavior		
	*β*	95% CI	*p* Value	*β*	95% CI	*p* Value
Sex						
Girls	Ref.			Ref.		
Boys	0.575	0.552–0.598	<0.001	−0.142	−0.163–(−0.121)	<0.001
Age						
12–13 years	Ref.			Ref.		
14–15 years	0.091	0.067–0.115	<0.001	0.281	0.259–0.303	<0.001
Survey year	−0.013	−0.018–(−0.007)	<0.001	−0.009	−0.014–(−0.004)	0.001
PPP category/capita, $						
Q1 100–4299	0.160	0.123, 0.197	<0.001	Ref.		
Q2 4300–7799	Ref.			0.569	0.535–0.603	<0.001
Q3 7800–15,999	0.316	0.280, 0.351	<0.001	0.653	0.618–0.688	<0.001
Q4 16,000–22,999	0.854	0.819, 0.888	<0.001	1.445	1.411–1.480	<0.001
Q5 > 23,000	0.571	0.537, 0.606	<0.001	1.532	1.497–1.566	<0.001

Linear regression models are adjusted for sex, age, survey year, and PPP/capita category simultaneously. PPP, purchasing power parity. *β*: linear regression coefficient. Sufficient physical activity: any physical activity ≥60 min/day; sedentary behavior: sitting time ≥2 h per day, except at school or for homework.

**Table 3:** Prevalence of sufficient physical activity and prevalence of low sedentary behavior in adolescents aged 12–15 years by sex, WHO region, and PPP category.

	Prevalence of Sufficient Physical Activity				Prevalence of Low Sedentary Behavior			
	Boys		Girls		Boys		Girls	
	N	Prevalence, %(95% CI)	N	Prevalence, %(95% CI)	N	Prevalence, %(95% CI)	N	Prevalence, %(95% CI)
WHO region								
Africa	1551	19.5 (14.9–24.2)	1162	12.4 (9.7–15.2)	5128	68.6 (62.2–75.0)	6058	65.6 (57.3–73.8)
America	5294	21.0 (19.3–22.6)	3610	12.4 (11.2–13.6)	14,357	57.4 (52.0–62.9)	14,423	52.5 (46.2–58.8)
East Mediterranean	4267	17.8 (14.7–20.9)	2684	11.0 (9.4–12.6)	15,117	66.8 (58.4–75.2)	15,048	65.1 (55.1–75.0)
Southeast Asia	1777	19.0 (12.1–25.9)	1890	14.7 (9.0–20.4)	7076	76.4 (66.1–86.7)	8938	78.0 (65.8–90.2)
Western Pacific	3897	17.1 (14.0–20.2)	2538	11.7 (9.5–13.9)	13,734	69.5 (64.2–74.8)	15,157	68.4 (61.9–74.9)
PPP category, $								
Q1 100–4299	2674	24.7 (21.8–27.8)	2433	19.6 (16.9–22.6)	12,267	83.7 (81.3–85.8)	12,463	84.3 (82.6–85.9)
Q2 4300–7799	3104	12.7 (11.4–14.1)	2343	8.4 (7.4–9.6)	13,012	70.4 (67.8–72.8)	15,787	68.7 (66.1–71.1)
Q3 7800–15,999	3149	16.0 (14.4–17.8)	2265	10.2 (9.0–11.6)	10,720	71.4 (69.0–73.8)	12,313	76.0 (73.9–77.9)
Q4 16,000–22,999	3902	19.5 (17.6–21.6)	2612	8.8 (7.8–10.0)	9377	50.6 (47.2–54.0)	9592	47.8 (45.2–50.4)
Q5 > 23,000	3957	19.9 (18.7–21.2)	2231	9.5 (8.7–10.3)	10,036	56.6 (54.6–58.5)	9469	53.4 (51.7–55.1)
Linear trend across quintiles		—		—		<0.001		<0.001

WHO, World Health Organization; PPP, purchasing power parity. Sufficient physical activity: physical activity ≥60 min/day; low sedentary behavior: sitting time <2 h/day except sitting time at school and for homework.

**Table 4:** Predictors of sufficient physical activity and low sedentary behavior in adolescents aged 12–15 years.

	Sufficient Physical Activity (≥60 min/Day)			Low Sedentary Behavior (≥2 h/Day Except Sitting Time at School and for Homework)		
	OR	95% CI	*p* Value	OR	95% CI	*p* Value
Sex						
Girls	Ref.			Ref.		
Boys	1.59	1.43–1.76	<0.001	0.95	0.88–1.03	0.229
Age						
12–13 years	Ref.			Ref.		
14–15 years	1.10	0.99–1.22	0.065	0.76	0.70–0.83	<0.001
Survey year	1.03	1.00–1.07	0.075	0.99	0.96–1.01	0.350
PPP/capita, $						
Q1 100–4799	2.44	2.08–2.85	<0.001	Ref.		
Q2 4800–7999	Ref.			0.44	0.38–0.51	<0.001
Q3 8000–12,299	1.31	1.15–1.50	<0.001	0.51	0.44–0.60	<0.001
Q4 12,300–18,999	1.39	1.20–1.61	<0.001	0.18	0.16–0.21	<0.001
Q5 > 19,000	1.52	1.34–1.73	<0.001	0.23	0.20–0.26	<0.001

Logistic regression models are adjusted for sex, age, survey year, and PPP/capita categories. PPP, purchasing power parity.

**Table 5:** Prevalence of combined categories of physical activity and sedentary behavior in adolescents aged 12–15 years by sex, WHO region, and PPP category.

	Sufficient Physical Activity				Insufficient Physical Activity			
	Low Sedentary Behavior		High Sedentary Behavior		Low Sedentary Behavior		High Sedentary Behavior	
	%(95% CI)		%(95% CI)		%(95% CI)		%(95% CI)	
	Boys	Girls	Boys	Girls	Boys	Girls	Boys	Girls
WHO Region								
Africa	12.1 (9.2–14.9)	7.3 (5.4–9.1)	7.1 (4.9–9.2)	4.7 (3.3–6.1)	55.9 (48.9–62.9)	57.8 (49.9–65.7)	24.1 (18.8–29.4)	29.5 (22.4–36.7)
America	11.2 (10.2–12.2)	6.1 (5.1–7.0)	9.6 (7.9–11.2)	6.2 (5.2–7.2)	46.1 (41.0–51.2)	46.3 (40.9–51.7)	32.7 (28.7–36.8)	41.1 (35.5–46.7)
East Mediterranean	10.5 (9.0–12.1)	6.6 (5.8–7.4)	7.0 (4.9–9.1)	4.1 (2.8–5.4)	56.0 (49.3–62.8)	58.3 (49.3–67.2)	26.0 (19.4–32.6)	30.8 (21.9–39.6)
Southeast Asia	14.0 (8.6–19.3)	11.9 (7.0–16.8)	4.5 (2.2–6.9)	2.4 (1.3–3.5)	61.6 (49.6–73.5)	64.8 (52.0–77.6)	18.7 (11.0–26.5)	19.3 (7.8–30.8)
Western Pacific	11.0 (8.8–13.1)	7.6 (6.0–9.2)	6.4 (4.6–8.2)	4.0 (3.1–4.9)	58.5 (53.5–63.6)	60.7 (54.8–66.5)	24.3 (19.8–28.8)	27.0 (22.2–31.9)
PPP/capita								
Q1 100–4299	12.0 (9.8–14.1)	9.6 (7.5–11.7)	4.4 (3.3–5.6)	2.7 (2.1–3.4)	65.4 (60.9–69.8)	67.6 (62.9–72.4)	17.4 (14.3–20.6)	19.0 (14.2–23.8)
Q2 4300–7799	10.0 (7.7–12.3)	6.5 (5.2–7.9)	5.4 (4.3–6.4)	3.6 (2.9–4.3)	60.4 (57.3–63.6)	63.5 (60.6–66.3)	24.0 (20.3–27.8)	26.0 (23.7–28.4)
Q3 7800–15,999	12.6 (10.5–14.7)	8.1 (6.7–9.5)	8.2 (6.3–10.1)	4.6 (3.5–5.7)	54.9 (49.8–59.9)	58.1 (51.4–64.9)	23.9 (21.3–26.5)	28.8 (24.1–33.5)
Q4 16,000–22,999	11.5 (10.2–12.9)	5.3 (4.5–6.2)	11.0 (9.6–12.4)	6.3 (4.9–7.8)	39.2 (35.0–43.4)	39.8 (34.3–45.3)	37.7 (33.9–41.5)	48.0 (42.6–53.4)
Q5 > 23,000	10.0 (8.7–11.4)	4.9 (3.8–6.0)	10.0 (8.6–11.4)	6.8 (5.3–8.3)	41.7 (38.0–45.3)	40.2 (34.6–45.9)	37.9 (35.0–40.8)	48.0 (43.1–52.8)
Linear trend across quintiles	<0.001	<0.001	<0.001	<0.001	<0.001	<0.001	<0.001	<0.001

WHO, World Health Organization; PPP, purchasing power parity. Sufficient physical activity: any physical activity ≥60 min/day; low sedentary behavior: sitting time <2 h/day except sitting time at school and for homework.

**Table 6:** Predictors of insufficient physical activity and of high sedentary behavior.

	Sufficient Physical Activity				Insufficient Physical Activity			
	Low Sedentary Behavior		High Sedentary Behavior		Low Sedentary Behavior		High Sedentary Behavior	
	OR (95% CI)	*p* Value	OR (95% CI)	*p* Value	OR (95% CI)	*p* Value	OR (95% CI)	*p* Value
Sex								
Girls	Ref.		Ref.		Ref.		Ref.	
Boys	1..41 (1.25–1.59)	<0.001	1.88 (1.62–2.18)	<0.001	0.84 (0.78–0.90)	<0.001	0.90 (0.84–0.97)	0.006
Age								
12–13 years	Ref.		Ref.		Ref.		Ref.	
14–15 years	1.06 (0.95–1.19)	0.314	1.17 (0.99–1.38)	0.060	0.78 (0.73–0.84)	<0.001	1.31 (1.21–1.41)	<0.001
Survey year	1.05 (1.00–1.09)	0.030	0.99 (0.95–1.04)	0.770	0.97 (0.95–1.00)	0.033	1.02 (0.99–1.04)	0.210
PPP category, $								
Q1 100–4299	Ref.		Ref.		Ref.		Ref.	
Q2 4300–7799	0.30 (0.28–0.36)	<0.001	1.08 (0.85–1.38)	0.529	0.91 (0.80–1.04)	0.174	2.53 (2.22–2.89)	<0.001
Q3 7800–15,999	0.42 (0.36–0.50)	<0.001	1.22 (0.96–1.55)	0.102	0.94 (0.82–1.09)	0.415	2.08 (1.82–2.39)	<0.001
Q4 16,000–22,999	0.34 (0.29–0.41)	<0.001	1.89 (1.51–2.38)	<0.001	0.38 (0.32–0.44)	<0.001	5.74 (4.93–6.67)	<0.001
Q5 > 23,000	0.40 (0.34–0.47)	<0.001	1.85 (1.51–2.25)	<0.001	0.45 (0.40–0.51)	<0.001	4.58 (4.08–5.14)	<0.001

Logistic regression models are adjusted for sex, age, survey year, and PPP/capita category simultaneously. PPP: purchasing power parity. Sufficient physical activity: any physical activity ≥60 min/day; low sedentary behavior: sitting for ≥2 h/day except sitting time at school and for homework.

## Supplementary Materials

The following are available online at https://www.mdpi.com/1660-4601/17/21/7752/s1, Table S1: Characteristics of young adolescents aged 12–15 years in the Global School–Based Student Health Survey across countries, 2009–2016., Table S2: Proportion of physical activity ≥1 h/day and/or sedentary behaviors ≤2 h/day in adolescents aged 12–15 years by country.
