# Supplementary material for: Physical Activity and Sedentary Behavior among Young Adolescents in 68 LMICs, and Their Relationships with National Economic Development
Source: Int J Environ Res Public Health. 2020 Oct 23;17(21):7752. doi: 10.3390/ijerph17217752 (PMC7660305; doi:10.3390/ijerph17217752)
Supplement: Supplementary file 1 [file ijerph-17-07752-s001.pdf]

Table S1. Characteristics of young adolescents aged 12–15 years in the Global School–Based Student Health Survey across countries, 2009–2016.

Country	Survey Years	Sample Size	Age, Years, Mean (SE)	Boys, %	Mean Days/Week of Physical Activity, Mean (SE)	Mean Hours/Day of Sedentary Behavior, Mean (SE)	Recommended Sufficient Physical Activity,%	Recommended Low Sedentary Time,%
**Africa**								
Algeria	2011	3410	13.63 (0.05)	45.6	2.49 (0.06)	2.06 (0.07)	14.9	73.2
Benin	2016	698	14.22 (0.07)	65.9	3.44 (0.24)	2.12 (0.16)	28.3	74.8
Ghana	2012	1309	13.86 (0.09)	48.3	1.96 (0.13)	1.67 (0.07)	10.2	81.0
Mauritania	2010	1199	14.17 (0.05)	53.0	1.79 (0.14)	2.73 (0.14)	10.9	60.8
Mauritius	2011	3032	13.83 (0.06)	48.7	3.10 (0.14)	2.63 (0.09)	19.2	60.9
Mozambique	2015	622	14.13 (0.15)	50.2	2.22 (0.22)	2.68 (0.17)	10.8	57.9
Namibia	2013	1864	14.09 (0.08)	42.5	1.95 (0.10)	2.77 (0.08)	14.2	63.1
Seychelles	2015	1921	13.51 (0.12)	47.6	2.54 (0.10)	3.35 (0.09)	17.5	51.0
Tanzania	2014	2499	13.63 (0.11)	47.0	2.76 (0.12)	1.71 (0.07)	20.9	79.9
**America**								
Antigua and Barbuda	2009	1106	13.91 (0.08)	51.4	2.94 (0.15)	3.64 (0.13)	22.8	45.6
Argentina	2012	20416	13.93 (0.04)	47.6	3.07 (0.04)	3.09 (0.04)	16.7	50.0
Bahamas	2013	1220	13.43 (0.11)	46.5	2.49 (0.17)	3.64 (0.09)	15.4	45.0
Barbados	2011	1419	14.10 (0.08)	50.3	2.77 (0.10)	3.95 (0.10)	19.1	34.9
Belize	2011	1501	13.56 (0.12)	48.1	2.73 (0.11)	2.63 (0.09)	20.0	63.3
Bolivia	2012	2692	14.05 (0.08)	49.9	2.61 (0.09)	1.91 (0.07)	14.0	75.4
British Virgin	2009	1156	13.54 (0.03)	43.8	2.65 (0.08)	3.85 (0.08)	18.7	39.6
Chile	2013	1284	13.79 (0.12)	48.8	3.03 (0.10)	3.20 (0.10)	15.6	47.2
Costa Rica	2009	2234	13.97 (0.02)	49.7	2.91 (0.09)	2.79 (0.07)	18.1	55.9
Curacao	2015	1352	13.95 (0.09)	48.4	2.26 (0.08)	3.72 (0.08)	12.0	41.3
El Salvador	2013	1569	13.99 (0.06)	51.1	2.20 (0.13)	2.35 (0.13)	12.6	64.7
Guatemala	2015	3304	13.90 (0.08)	51.1	2.10 (0.12)	1.83 (0.13)	11.3	77.1
Guyana	2010	1898	14.05 (0.05)	48.3	2.14 (0.17)	2.55 (0.11)	14.9	64.2
Honduras	2012	1417	13.57 (0.07)	46.0	2.25 (0.09)	2.16 (0.06)	15.1	69.6
Peru	2010	2335	14.11 (0.04)	50.0	2.71 (0.09)	2.07 (0.08)	15.1	71.2
Saint Kitts and Nevis	2011	1423	14.08 (0.02)	43.2	2.57 (0.07)	3.81 (0.07)	18.1	40.8
Suriname	2009	1004	13.95 (0.07)	44.9	2.70 (0.13)	2.64 (0.11)	19.8	59.6

Trinidad and Tobago	2011	2219	13.59 (0.07)	49.2	2.88 (0.13)	2.87 (0.07)	20.5	55.9
Uruguay	2012	2799	14.09 (0.04)	46.0	3.01 (0.08)	3.32 (0.06)	16.4	41.6
**East Mediterranean**								
Afghanistan	2014	1399	14.06 (0.06)	53.6	2.21 (0.12)	1.84 (0.14)	10.0	76.6
Bahrain	2016	5319	13.65 (0.08)	50.2	3.03 (0.09)	3.66 (0.10)	21.0	43.4
Egypt	2011	2279	13.51 (0.06)	49.1	2.06 (0.14)	2.18 (0.13)	12.9	72.6
Iraq	2012	1451	13.89 (0.07)	54.6	2.13 (0.10)	1.96 (0.07)	14.8	74.1
Kuwait	2015	1803	14.06 (0.11)	50.8	2.87 (0.11)	3.97 (0.17)	17.1	36.8
Lebanon	2011	1869	13.73 (0.10)	46.5	3.30 (0.08)	2.93 (0.08)	23.4	52.8
Morocco	2010	2319	13.66 (0.03)	52.7	2.23 (0.06)	1.98 (0.06)	12.7	74.2
Oman	2015	1616	14.19 (0.02)	43.9	2.37 (0.06)	2.66 (0.06)	13.3	62.3
Pakistan	2009	4893	14.12 (0.04)	60.8	1.65 (0.19)	1.11 (0.04)	11.6	91.8
Palestine	2010	13161	13.79 (0.04)	48.6	2.33 (0.06)	2.27 (0.04)	16.3	67.2
Qatar	2011	1538	13.41 (0.08)	47.0	2.09 (0.11)	3.24 (0.11)	10.6	51.1
Sudan	2012	1334	14.17 (0.07)	52.3	1.77 (0.11)	1.65 (0.11)	7.8	80.4
Syria	2010	2867	13.59 (0.08)	51.2	1.86 (0.10)	1.86 (0.08)	11.3	74.8
United Arab Emirates	2010	2190	13.97 (0.06)	39.0	2.90 (0.11)	3.16 (0.10)	17.3	49.0
Yemen	2014	1471	13.83 (0.07)	56.1	1.93 (0.14)	1.65 (0.13)	13.2	80.9
**Southeast Asia**								
Bangladesh	2014	2618	13.95 (0.08)	63.6	4.05 (0.15)	1.52 (0.09)	41.4	85.0
Indonesia	2015	8512	13.45 (0.05)	49.3	1.90 (0.07)	1.91 (0.06)	12.0	75.5
Nepal	2015	4438	13.76 (0.04)	48.1	1.87 (0.18)	1.16 (0.05)	14.7	90.2
Thailand	2015	4012	13.67 (0.06)	49.0	2.38 (0.07)	3.16 (0.11)	12.0	49.4
Timor–Leste	2015	1530	14.09 (0.06)	46.1	1.63 (0.07)	1.55 (0.07)	8.1	84.3
**Western Pacific**								
Brunei Darussalam	2014	1772	13.96 (0.06)	47.9	2.65 (0.07)	3.27 (0.08)	11.6	45.5
Cambodia	2013	1775	14.05 (0.07)	48.4	1.42 (0.05)	1.22 (0.06)	6.5	89.7
Cook	2015	360	14.21 (0.12)	48.6	3.27 (0.19)	2.62 (0.12)	16.1	57.9
Fiji	2016	1429	14.41 (0.04)	48.3	2.92 (0.10)	2.16 (0.10)	19.6	71.7
French Polynesia	2015	1798	13.69 (0.10)	48.7	3.30 (0.10)	2.65 (0.08)	16.8	60.5
Kiribati	2011	1322	14.04 (0.08)	45.3	2.53 (0.08)	1.46 (0.05)	17.2	85.6
Laos	2015	1629	14.48 (0.04)	47.9	2.31 (0.08)	1.65 (0.07)	16.3	80.9
Malaysia	2012	16081	13.97 (0.02)	49.5	2.57 (0.05)	2.71 (0.04)	13.8	57.3

Mongolia	2013	3643	13.67 (0.05)	49.2	3.50 (0.06)	2.56 (0.08)	26.8	60.2
Nauru	2011	309	13.72 (0.06)	38.5	1.55 (0.13)	2.78 (0.17)	10.4	67.0
Niue	2010	78	13.42 (0.14)	56.4	2.79 (0.28)	2.35 (0.29)	12.8	71.8
Philippines	2015	6028	13.88 (0.05)	48.0	1.46 (0.09)	2.16 (0.10)	7.4	69.3
Samoa	2011	1838	14.03 (0.06)	46.4	2.39 (0.06)	2.61 (0.08)	12.6	62.8
Solomon	2011	872	14.08 (0.07)	51.9	2.73 (0.12)	2.10 (0.13)	16.8	72.8
Tokelau	2014	81	13.42 (0.05)	56.8	3.03 (0.56)	2.98 (0.05)	26.1	56.2
Tonga	2010	1908	14.06 (0.07)	50.5	2.40 (0.09)	2.34 (0.08)	13.8	70.7
Tuvalu	2013	631	13.34 (0.04)	49.1	1.69 (0.10)	1.54 (0.07)	11.7	84.6
Vanuatu	2011	832	13.45 (0.12)	49.3	3.28 (0.41)	1.84 (0.13)	10.6	81.2
Vietnam	2013	1731	14.50 (0.04)	46.8	2.20 (0.08)	2.31 (0.08)	12.9	65.1
Wallis and Futuna	2015	660	13.74 (0.14)	48.7	2.66 (0.14)	2.39 (0.12)	14.2	66.4
**Total**		180298	13.88 (0.03)	48.3	2.48 (0.06)	2.48 (0.09)	15.3	64.6

Table S2. Proportion of physical activity ≥1 h/day and/or sedentary behaviors ≤2 h/day in adolescents aged 12–15 years by country.

Country	Physical activity ≥ 1 h per day		Physical activity < 1 h per day	
	Sedentary behaviors ≤ 2 h per day, %	Sedentary behaviors > 2 h per day, %	Sedentary behaviors ≤ 2 h per day, %	Sedentary behaviors > 2 h per day, %
**Africa**				
Algeria	9.6 (8.3–11.1)	5.3 (4.5–6.2)	63.6 (61.2–66.0)	21.5 (18.8–24.5)
Benin	19.9 (14.2–27.2)	8.4 (5.9–11.7)	54.9 (48.1–61.5)	16.9 (13.1–21.5)
Ghana	7.7 (6.5–9.1)	2.5 (1.7–3.7)	73.3 (70.3–76.1)	16.5 (14.1–19.2)
Mauritania	6.1 (4.5–8.2)	4.8 (3.3–6.8)	54.7 (49.4–59.9)	34.4 (29.3–40.0)
Mauritius	11.8 (9.9–14.0)	7.4 (6.1–9.1)	49.1 (46.3–52.0)	31.6 (28.2–35.3)
Mozambique	5.7 (3.3–9.9)	5.1 (2.9–8.7)	52.2 (44.3–60.0)	37.0 (30.9–43.5)
Namibia	7.5 (6.0–9.4)	6.6 (5.6–7.8)	55.6 (52.6–58.5)	30.2 (27.3–33.3)
Seychelles	8.2 (6.8–9.9)	9.2 (7.8–10.8)	42.7 (40.0–45.6)	39.8 (36.9–42.7)
Tanzania	15.7 (12.9–19.0)	5.2 (4.0–6.6)	64.2 (59.5–68.6)	14.9 (12.8–17.4)
Pooled estimates	9.5 (7.7–11.3)	6.0 (4.6–7.3)	56.8 (49.7–63.8)	26.9 (20.4–33.3)
I^2 (%)	86.4	88.8	97.3	97.2
**America**				
Antigua and Barbuda	8.6 (6.6–10.9)	14.2 (11.5–17.5)	37.1 (32.5–41.9)	40.1 (36.4–44.0)
Argentina	9.0 (8.2–9.9)	7.7 (7.0–8.5)	41.0 (39.6–42.3)	42.3 (40.8–43.8)
Bahamas	6.2 (4.8–8.1)	9.2 (7.5–11.3)	38.7 (35.0–42.6)	45.8 (42.3–49.4)
Barbados	6.2 (5.0–7.8)	12.8 (11.0–15.0)	28.7 (25.4–32.2)	52.2 (49.3–55.2)
Belize	11.7 (9.7–14.1)	8.2 (6.8–9.9)	51.5 (47.3–55.8)	28.5 (25.1–32.2)
Bolivia	10.5 (8.8–12.5)	3.5 (2.7–4.5)	64.9 (61.8–67.8)	21.2 (18.9–23.6)
British Virgin	6.8 (5.3–8.3)	11.9 (10.0–13.8)	32.8 (30.1–35.5)	48.5 (45.6–51.4)
Chile	8.6 (7.2–10.2)	7.0 (5.3–9.1)	38.6 (35.0–42.3)	45.9 (41.6–50.1)
Costa Rica	10.5 (9.1–12.1)	7.6 (6.4–8.9)	45.5 (42.0–48.9)	36.5 (33.5–39.5)
Curacao	4.6 (3.5–5.9)	7.4 (6.3–8.8)	36.7 (33.7–39.9)	51.2 (47.6–54.9)
El Salvador	7.4 (6.0–9.1)	5.2 (3.8–7.1)	57.3 (51.3–63.2)	30.1 (25.8–34.7)
Guatemala	8.0 (5.7–11.2)	3.3 (2.4–4.4)	69.1 (62.8–74.8)	19.6 (15.2–24.9)
Guyana	7.9 (6.2–10.1)	7.0 (5.1–9.6)	56.3 (51.0–61.5)	28.8 (25.4–32.4)
Honduras	9.5 (8.0–11.1)	5.7 (4.6–6.9)	60.1 (57.0–63.2)	24.7 (22.6–27.0)

Peru	11.0 (9.5–12.7)	4.1 (3.3–5.1)	60.2 (56.9–63.3)	24.7 (21.9–27.9)
Saint Kitts and Nevis	7.2 (5.9–8.5)	11.0 (9.4–12.6)	33.7 (31.2–36.2)	48.2 (45.6–50.8)
Suriname	10.5 (8.9–12.5)	9.2 (7.4–11.5)	49.0 (43.9–54.2)	31.2 (27.0–35.7)
Trinidad and Tobago	10.8 (9.0–12.9)	9.7 (7.9–11.8)	45.1 (42.2–47.9)	34.5 (31.2–37.9)
Uruguay	8.4 (7.4–9.5)	8.1 (6.7–9.6)	33.3 (30.7–36.0)	50.3 (47.7–52.9)
Pooled estimates	8.5 (7.7–9.4)	7.9 (6.7–9.2)	46.2 (41.2–51.1)	37.1 (32.2–42.0)
I^2 (%)	84.3	94.0	97.9	98.1
**Eastern Mediterranean**				
Afghanistan	7.6 (5.6–10.2)	2.4 (1.5–3.8)	69.0 (62.6–74.8)	21.0 (15.7–27.4)
Bahrain	10.1 (8.3–12.3)	10.8 (9.7–12.1)	33.3 (31.2–35.5)	45.7 (41.5–50.0)
Egypt	7.0 (5.5–8.9)	5.9 (3.7–9.2)	65.6 (60.0–70.8)	21.5 (18.0–25.6)
Iraq	10.4 (8.2–13.3)	4.4 (3.3–5.7)	63.7 (60.0–67.2)	21.5 (18.5–24.8)
Kuwait	6.6 (4.8–8.9)	10.5 (8.9–12.4)	30.2 (26.4–34.4)	52.7 (47.4–57.9)
Lebanon	12.6 (10.7–14.9)	10.8 (8.8–13.1)	40.1 (36.6–43.8)	36.4 (32.5–40.6)
Morocco	8.5 (7.0–10.2)	4.2 (3.6–5.0)	65.8 (63.6–67.9)	21.5 (19.3–23.9)
Oman	7.7 (6.4–9.0)	5.6 (4.5–6.7)	54.6 (52.2–57.0)	32.1 (29.8–34.4)
Pakistan	10.3 (6.1–16.8)	1.3 (0.8–2.1)	81.6 (76.0–86.1)	6.9 (5.8–8.1)
Palestine	10.3 (9.4–11.3)	6.0 (5.3–6.8)	56.9 (55.0–58.7)	26.8 (25.2–28.5)
Qatar	4.7 (3.1–7.1)	5.9 (4.5–7.7)	46.4 (42.6–50.2)	43.1 (39.2–47.1)
Sudan	5.5 (3.8–8.0)	2.2 (1.3–3.9)	74.9 (71.0–78.3)	17.4 (14.5–20.7)
Syria	8.1 (6.3–10.3)	3.2 (2.5–4.2)	66.7 (61.9–71.3)	22.0 (17.7–26.9)
United Arab Emirates	8.8 (7.2–10.8)	8.5 (7.1–10.1)	40.1 (36.8–43.5)	42.5 (39.2–45.9)
Yemen	10.9 (8.3–14.1)	2.3 (1.3–4.0)	70.0 (64.3–75.2)	16.8 (13.2–21.1)
Pooled estimates	8.5 (7.4–9.6)	5.5 (4.0–7.0)	57.2 (49.8–64.6)	28.5 (21.6–35.4)
I^2 (%)	79.0	96.5	98.8	98.9
**South-East Asia**				
Bangladesh	35.0 (30.5–39.8)	6.3 (4.6–8.6)	50.0 (45.2–54.8)	8.6 (6.2–12.0)
Indonesia	8.5 (7.2–10.0)	3.5 (3.0–4.1)	67.0 (64.2–69.8)	21.0 (18.8–23.4)
Nepal	13.6 (9.5–19.0)	1.1 (0.7–1.6)	76.6 (71.1–81.3)	8.8 (6.9–11.1)
Thailand	5.9 (4.7–7.4)	6.1 (5.0–7.3)	43.5 (39.3–47.8)	44.5 (40.1–49.1)
Timor–Leste	6.3 (4.8–8.2)	1.8 (1.2–2.6)	78.1 (75.0–80.9)	13.9 (11.5–16.6)
Pooled estimates	13.3 (7.9–18.8)	3.6 (1.9–5.4)	63.1 (50.4–75.7)	19.2 (9.8–28.6)

I^2 (%)	97.2	96.3	98.2	98.3
**Western Pacific**				
Brunei Darussalam	5.7 (4.7–6.9)	5.9 (4.7–7.4)	39.9 (36.9–42.8)	48.5 (45.4–51.6)
Cambodia	5.7 (4.3–7.6)	0.8 (0.4–1.5)	84.0 (79.7–87.5)	9.5 (7.2–12.5)
Cook	10.4 (7.1–14.9)	5.8 (3.4–9.6)	47.6 (42.7–52.5)	36.3 (31.4–41.5)
Fiji	13.6 (11.8–15.6)	6.0 (4.4–8.2)	58.1 (54.8–61.3)	22.3 (19.1–25.8)
French Polynesia	10.0 (8.6–11.6)	6.9 (5.9–8.0)	50.6 (47.9–53.3)	32.6 (29.8–35.5)
Kiribati	14.1 (12.4–16.0)	3.0 (2.3–4.0)	71.4 (68.5–74.2)	11.4 (9.6–13.4)
Laos	12.4 (10.4–14.6)	3.9 (3.0–5.1)	68.5 (64.9–72.0)	15.1 (12.5–18.2)
Malaysia	7.8 (7.1–8.5)	6.0 (5.4–6.6)	49.6 (47.8–51.3)	36.7 (35.0–38.4)
Mongolia	15.5 (14.3–16.9)	11.3 (10.2–12.5)	44.6 (41.6–47.8)	28.5 (25.2–32.1)
Nauru	4.9 (2.5–7.3)	5.5 (3.0–8.0)	62.1 (56.7–67.5)	27.5 (22.5–32.5)
Niue	10.3 (3.6–17.0)	2.6 (0–6.1)	61.5 (50.7–72.3)	25.6 (15.9–35.3)
Philippines	3.4 (2.8–4.1)	4.0 (2.9–5.4)	65.9 (61.9–69.7)	26.7 (24.0–29.7)
Samoa	8.0 (6.5–9.9)	4.5 (3.7–5.6)	54.8 (51.7–57.9)	32.6 (30.0–35.4)
Solomon	11.1 (9.5–13.1)	5.6 (3.5–9.1)	61.7 (56.7–66.5)	21.5 (18.8–24.6)
Tokelau	13.5 (5.8–28.3)	12.6 (7.9–19.4)	42.6 (27.3–59.5)	31.3 (25.7–37.5)
Tonga	8.9 (7.4–10.6)	4.9 (3.9–6.1)	61.9 (59.2–64.4)	24.4 (22.0–26.9)
Tuvalu	8.6 (6.4–10.8)	3.2 (1.8–4.6)	76.1 (72.8–79.4)	12.2 (9.6–14.8)
Vanuatu	7.6 (4.9–11.6)	3.0 (1.8–4.9)	73.6 (67.3–79.0)	15.8 (12.4–19.9)
Vietnam	8.6 (7.0–10.4)	4.3 (3.4–5.6)	56.5 (52.3–60.6)	30.6 (26.9–34.5)
Wallis and Futuna	8.7 (6.2–12.1)	5.5 (3.8–7.9)	57.7 (52.6–62.6)	28.1 (23.8–33.0)
Pooled estimates	9.3 (7.5–11.0)	5.1 (3.9–6.2)	59.7 (54.5–64.9)	25.8 (20.9–30.7)
I^2 (%)	96.1	94.9	97.7	98.1
**Total**				
Pooled estimates	9.1 (8.4–9.8)	6.0 (5.3–6.7)	55.2 (51.9–58.4)	29.2 (26.1–32.4)
I^2 (%)	92.5	96.1	98.5	98.8
